# Portal Vein Thrombosis Is Associated with an Increased Incidence of Depression and Anxiety Disorders

**DOI:** 10.3390/jcm10235689

**Published:** 2021-12-02

**Authors:** Simon Johannes Gairing, Peter Robert Galle, Jörn M. Schattenberg, Karel Kostev, Christian Labenz

**Affiliations:** 1 Department of Internal Medicine I, University Medical Center of the Johannes Gutenberg-University, 55131 Mainz, Germany; simonjohannes.gairing@unimedizin-mainz.de (S.J.G.); peter.galle@unimedizin-mainz.de (P.R.G.); joern.schattenberg@unimedizin-mainz.de (J.M.S.); 2 Cirrhosis Center Mainz (CCM), University Medical Center of the Johannes Gutenberg-University, 55131 Mainz, Germany; 3 Epidemiology, IQVIA, 60549 Frankfurt am Main, Germany; Karel.Kostev@iqvia.com

**Keywords:** portal vein thrombosis, liver cirrhosis, depression, anxiety disorders

## Abstract

Portal vein thrombosis (PVT) is a severe disease that adversely affects patients’ well-being. Data on the influence of PVT on the occurrence of depression or anxiety disorders are lacking. This study aimed to explore the impact of PVT on the incidence of depression and anxiety disorders diagnoses in a large German primary care cohort over a ten-year period. Patients with PVT were matched to non-PVT individuals by age, sex, yearly consultation frequency, index year and comorbidities in a 1:5 ratio. The primary outcome of the study was the incidence of depression and anxiety disorders. The relationship between PVT and both depression and anxiety disorders was investigated using Cox regression models. We compared 547 patients with PVT with 2735 matched individuals without PVT. Within 5 years of the index date, 17.4% of patients with PVT and 9.3% of non-PVT individuals were diagnosed with depression (*p* < 0.001). Anxiety disorders were diagnosed in 5.5% and 3.0% of patients with PVT and non-PVT individuals, respectively (*p* = 0.002). On regression analyses, PVT was positively associated with incident depression (HR 2.01, 95% CI 1.53–2.64, *p* < 0.001) as well as anxiety disorders (HR 2.16, 95% CI 1.35–3.46, *p* = 0.001). Regarding depression, this association remained significant in women as well as in men. There was no association between PVT and the incidence of anxiety disorders in women. In conclusion, PVT is associated with the development of depression and anxiety disorders. However, further prospective studies are needed to confirm our findings before definitive recommendations can be made.

## 1. Introduction

Portal vein thrombosis (PVT) is a frequent and often severe complication in patients with liver cirrhosis, with a prevalence ranging from 10% to 25% [1,2,3]. Non-cirrhotic PVT is associated with pro-thrombotic disorders such as myeloproliferative diseases or antiphospholipid syndrome [4]. Depending on whether the PVT occurs acutely or chronically, clinical presentation ranges from abdominal symptoms to portal hypertension-driven complications such as ascites or esophageal varices. In particular, the latter complications are comparable to the disease burden caused by liver cirrhosis-related portal hypertension [5].

Mood disorders including depression and anxiety disorders lead to poorer health-related quality of life and functional disability [6]. Globally, the prevalence of major depression is around 5% among adults [7]. According to the European Health Interview Survey 2, which includes data from 28 European Union member states and was collected between 2013–2015, the prevalence of depressive symptoms in Germany was significantly higher with 9.2% compared to the European Union average of 6.6% [8]. In addition to its adverse impact on quality of life, depression represents an increasingly important public health burden [4,5].

In patients with liver cirrhosis, mood disorders such as depression or anxiety disorders represent a major burden. A recent multicenter prospective study found a prevalence of depression of 18% among cirrhotic outpatients [9]. Nardelli et al. even reported a prevalence of depression, state-anxiety and trait-anxiety of 57%, 27% and 28%, respectively, resulting in poorer health-related quality of life [10]. In addition, the incidence of depression increases with the severity of liver cirrhosis, and depressive symptoms in turn increase the risk of hospitalization in patients with liver cirrhosis [11,12]. However, data on the risk of depression or anxiety disorders in patients with PVT are currently lacking.

There is a well-established bi-directional relationship between cardiovascular diseases (CVDs) and mood disorders such as depression [13,14]. Likewise, depression increases the risk of developing venous thromboembolism [15]. This is explained by pathophysiological mechanisms linking altered platelet aggregation, hyperactivations of the thrombosis cascade and endothelial dysfunctions to hypothalamic-pituitary-adrenocortical dysfunctions, which may result in depression [16]. In addition, recent studies suggest an association between systemic inflammation, which has already been linked to the occurrence of depression [17], and the risk of developing PVT [2,18]. Given this evidence, we hypothesized that PVT may be associated with a higher risk for incidental depression or anxiety disorders. Therefore, this study aimed to investigate the impact of PVT on the incidence of depression and anxiety disorders over a ten-year period in a large German primary care cohort.

## 2. Methods

### 2.1. Database

This study was based on data from the Disease Analyzer database (IQVIA), which compiles drug prescriptions, diagnoses and basic medical and demographic data obtained directly and in anonymous format from computer systems used in the practices of general practitioners and specialists [19]. Diagnoses (International Classification of Diseases, 10th revision (ICD–10)), prescriptions (European Pharmaceutical Market Research Association (EPhMRA) Anatomica Therapeutic Chemical Classification (ATC) system) and the quality of reported data are monitored by IQVIA based on a number of criteria (e.g., completeness of documentation and linkage between diagnoses and prescriptions). The sampling method for the Disease Analyzer database is based on statistics from all doctors in Germany. These statistics are used to determine the panel composition according to the following strata: region, community size category and age of physician [19].

### 2.2. Study Population

This study included adult (≥18 years) patients who received a first diagnosis of PVT (ICD–10: I81) in one of 1274 general practices in Germany between January 2000 and December 2019 (index date). Patients had to have a follow-up time of at least 6 months after the index date. Patients with depression (ICD–10: F32, F33), bipolar disorder (ICD–10: F30) and anxiety disorders (ICD–10: F41) diagnoses prior to or on the index date were excluded. After applying similar inclusion criteria, patients without PVT were matched to those with PVT. Greedy nearest-neighbor propensity score matching (1:5) based on sex, age, co-diagnoses documented within 12 months prior to the index date (cancer (ICD–10: C01–C97), obesity (ICD–10: C66), liver fibrosis and cirrhosis (ICD10: K70.3, K74) or chronic hepatitis (ICD10: K73), thrombophlebitis (ICD–10: I80) and varicose (ICD10: I83–I85)), and yearly consultation frequency was performed. Prior to the matching, these co-diagnoses were more frequently among patients with PVT, and were associated with depression risk in feasibility analyses. As PVT patients had a much higher general practitioner (GP) consultation frequency, and higher consultation frequency can increase the probability that outcome diagnoses (depression, anxiety disorders) will be documented, we included consultation frequency per year into the matching process.

**Figure 1:**
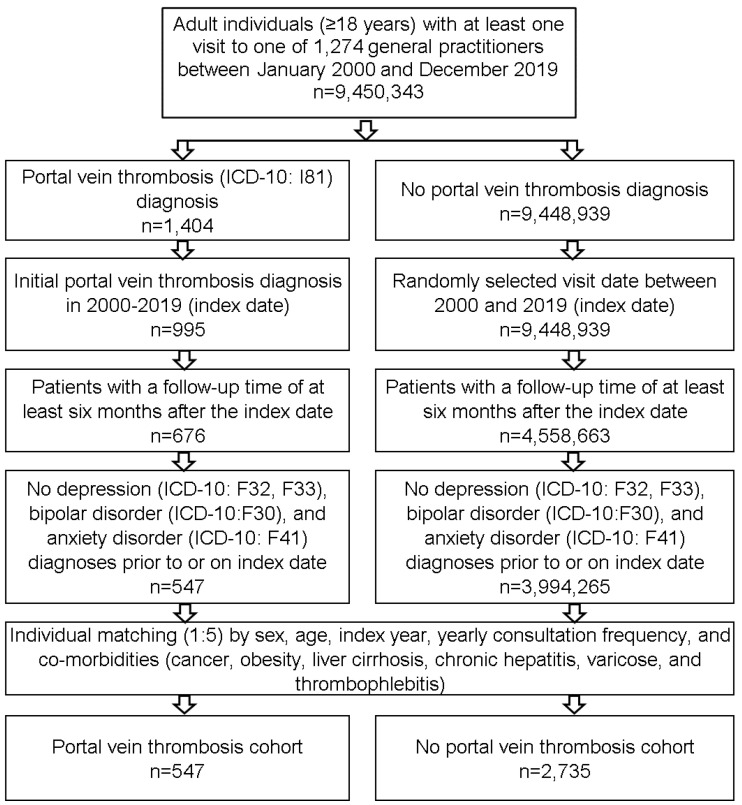
Selection of study patients.

For individuals without PVT, the index date corresponded to a randomly selected visit date between January 2000 and December 2019 (Figure 1).

### 2.3. Ethics Approval

This study was conducted according to the ethical guidelines of the 1964 Declaration of Helsinki (amended, 2013). We used anonymous electronic medical records for research purposes with no directly identifiable data. Accordingly, this study did not collect informed consent from individual patients and according to German regulations no ethical approval was needed. Anonymized data were analyzed as aggregates with no protected health information available.

### 2.4. Study Outcome and Statistical Analyses

The study outcome was the cumulative incidence of depression (ICD–10: F32, F33) and anxiety disorders (ICD–10: F41) as functions of PVT. After 1:5 matching, the age, sex, comorbidities and yearly consultation frequency of PVT patients were compared with those without PVT using McNemar tests for categorical variables and a paired Wilcoxon signed-rank test for continuous variables. Kaplan-Meier curves were used to compare the incidence of depression and anxiety disorders in the five years following the index year between patients with and without portal vein thrombosis. As there was no information on mortality, dead patients were considered lost to follow-up. Finally, the relationship between portal vein thrombosis, and both depression and anxiety disorders in the overall sample and separately in women and men was investigated using Cox regression models. The results of the Cox regression analyses are presented as Hazard Ratios (HRs) with 95% CIs. *p*-values < 0.05 were considered statistically significant. All analyses were performed using SAS 9.4 (SAS Institute, Cary, NC, USA).

## 3. Results

### 3.1. Baseline Characteristics

This study included 547 patients with PVT and 2735 controls without PVT. After 1:5 matching, there were no significant differences in age (57.3 versus 57.5 years), sex (38.9% versus 38.7% female), comorbidities and consultation frequency between the PVT cohort and non-PVT individuals. (Table 1).

### 3.2. Incidence of Depression in Patients with and without PVT

**Figure 2:**
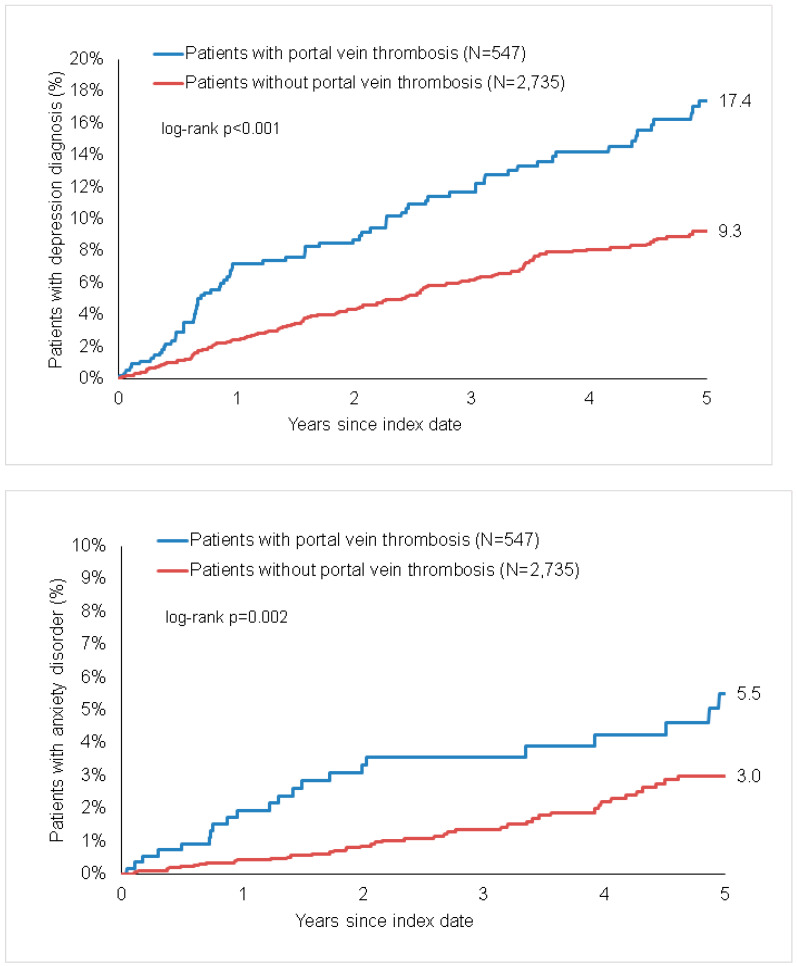
Kaplan-Meier curves for time to diagnosis of depression (**upper panel**) and anxiety disorders (**lower panel**) in patients with and without portal vein thrombosis.

The cumulative incidence of depression (17.4% versus 9.3%, log-rank *p* < 0.001) was significantly higher in patients with PVT than in those without PVT (Figure 2). The results of the Cox regression analyses are displayed in Table 2. PVT was positively associated with depression (HR = 2.01, 95% CI = 1.53–2.64). In subgroup analyses, this association between PVT and depression remained significant in both women and men, although the association was more pronounced in men than in women.

### 3.3. Incidence of Anxiety Disorders in Patients with and without PVT

The cumulative incidence of anxiety disorders (5.5% versus 3.9%, log-rank *p* = 0.002) was significantly higher in patients with PVT than in those without PVT (Figure 2). The results of the Cox regression analyses are displayed in Table 2. PVT was positively associated with anxiety disorders (HR = 2.16, 95% CI = 1.35–3.46). In subgroup analyses, there was a strong association between PVT and the incidence of anxiety disorders in men, while there was only a trend for an association in women.

## 4. Discussion

In this study, we observed a high coded incidence particularly of depression in patients with PVT. Additionally, we found that PVT was associated with a significant increase in incident depression as well as anxiety disorders compared to individuals without PVT after careful matching. The association between PVT and incident depression or anxiety disorders was more pronounced in men than in women.

To date, this association was unknown. In contrast, the relationship between depression and liver cirrhosis has already been reported: cirrhotic patients often suffer from depression, with a prevalence of up to 57%, and severity of liver disease increases the incidence of depression [10,11]. Regarding CVDs, a bi-directional relationship with depression is known: patients with CVD are more likely to suffer from depression, and depressed patients have a higher risk of CVD and subsequently higher mortality [20]. Moreover, there is mounting evidence for a bi-directional relationship between depression and the risk of venous thromboembolism [15].

It is an interesting finding that the association between PVT and the incidence of depression or anxiety disorders was stronger in men than in women. This contrasts with studies that examined the incidence of depression at the population level in Germany [21]. However, there are also data indicating that in patients with severe diseases, e.g., cancer, men may be at higher risk for depression or anxiety disorders [22]. This could be due to differences in coping strategies and how men and women deal with a serious diagnosis. However, this needs to be investigated in detail in future studies.

Due to our current study design, we can only detect associations and are therefore unable to investigate the pathophysiology behind our findings. Therefore, we can only hypothesize regarding possible explanations for the robust association between PVT and depression as well as anxiety disorders in our study. First, it is a well-known fact that (chronic) physical illness in general leads to an increased risk of depression [23,24]. Second, PVT may present with abdominal pain. Pain, in turn, is an important risk factor for depression [25]. Importantly, both depression and anxiety disorders exacerbate the perception of pain [25], leading to a continuous vicious cycle. Third, a subset of patients with PVT are treated with permanent anticoagulation (especially those with PVT without liver cirrhosis), which in turn leads to an increased risk of bleeding. Additionally, patients with PVT often suffer from additional severe and often rare diseases. These factors may lead to psychological distress that in turn promotes the development of depression or anxiety disorders. Unfortunately, due to our study design, we cannot prove these hypotheses in our current analysis.

Our current findings indicating a robust association between PVT and depression as well as anxiety disorders, are of pivotal clinical importance. The co-occurrence of long-term physical illness, such as PVT, and mental disorders have a negative impact on quality of life and treatment outcomes, and are also a major burden for healthcare systems by increasing costs [23]. Our data highlight that screening for depression and anxiety disorders not only by specialists but also by primary care physicians may be warranted. In turn, this may not only improve patients’ mental health and well-being, but also their prognosis on the long term.

The major strengths of our study are the comparatively large patient cohorts, the careful matching and the long follow-up period. However, there are also some limitations —mostly due to the study design—that have to be acknowledged. First, we are only able to detect associations and cannot prove causality. Additionally, the underlying etiology of PVT can be very heterogeneous (e.g., liver cirrhosis, systemic pro-thrombotic diseases such as myeloproliferative diseases) and these comorbidities may influence the risk of incident depression or anxiety disorders. Based on our matching criteria, we are confident to reduce this bias to a minimum. However, due to the small sample sizes in some subgroups (e.g., patients with liver cirrhosis), sensitivity analyses have to be interpreted with caution. Furthermore, unknown potentially confounding baseline characteristics can only be controlled for by randomization and not by retrospective matching. We also have to acknowledge that our study has the weaknesses inherent to all database analysis, as it relies on ICD–10 codes for establishing diagnoses. Thus, the extent of underlying misclassification related to undercoding or miscoding of diagnoses cannot be assessed. Last, the Disease Analyzer database does not capture detailed laboratory values nor information on disease stages, education, or employment status. These factors may influence the incidence of depression or anxiety disorders and should be kept in mind when interpreting our findings.

In conclusion, this study demonstrates that the coded incidence of especially depression is high in patients with PVT. Additionally, we found an association between PVT and incident depression as well as anxiety disorders when compared to individuals without PVT. However, further prospective studies are needed to confirm our findings before definitive recommendations can be made.

## Figures and Tables

**Table 1:** Baseline characteristics of study patients after 1:5 matching.

Variable	Patients with Portal Vein Thrombosis (*n* = 547)	Patients without Portal Vein Thrombosis (*n* = 2735)	*p* Value
Women	38.9	38.7	0.907
Men	61.1	61.3	
Mean age in years (standard deviation)	57.3 (16.1)	57.5 (16.0)	0.898
Age ≤50 years	32.2	32.3	0.998
Age 51–60 years	22.7	22.3	
Age 61–70 years	21.2	21.1	
Age >70 years	24.0	24.2	
Mean number of consultations per year	5.3 (7.4)	4.9 (6.7)	0.411
Diagnoses documented within 12 months prior to the index date			
Cancer	21.9	21.3	0.727
Obesity	10.6	10.3	0.835
Liver cirrhosis or chronic hepatitis	18.8	16.4	0.125
Thrombophlebitis	16.8	15.4	0.406
Varicose	27.6	25.4	0.275

Data are percentages unless otherwise specified.

**Table 2:** Association between portal vein thrombosis and the 5-year-incidence of depression and anxiety disorders.

	Depression		Anxiety Disorder	
	Hazard Ratio (95% CI)	*p* Value	Hazard Ratio (95% CI)	*p* Value
Overall	2.01 (1.53–2.64)	<0.001	2.16 (1.35–3.46)	0.001
Women	1.62 (1.30–2.54)	0.017	1.82 (0.89–3.73)	0.101
Men	2.45 (1.68–3.57)	<0.001	2.42 (1.30–4.54)	0.005
Patients with liver cirrhosis	2.27 (1.27–4.04)	0.006	2.31 (0.55–9.68)	0.253
Patients without liver cirrhosis	1.93 (1.42–2.63)	<0.001	2.18 (1.26–3.77)	0.006

## Data Availability

The data that support the findings of this study are available from K.K. and IQVIA. Restrictions apply to the availability of these data, which were used under license for this study. Data are available from K.K. with the permission of IQVIA.

## Abbreviations

Portal vein thrombosis	PVT
cardiovascular diseases	CVDs
Disease Analyzer database	IQVIA
International Classification of Diseases, 10th revision	ICD–10
European Pharmaceutical Market Research Association	EPhMRA
Anatomical Therapeutic Chemical Classification	ATC
general practitioner	GP
Hazard Ratio	HR
gamma-aminobutyric acid	GABA
brain-derived neurotrophic factor	BDNF
